# The Phosphatidylcholine Transfer Protein Stard7 is Required for Mitochondrial and Epithelial Cell Homeostasis

**DOI:** 10.1038/srep46416

**Published:** 2017-04-12

**Authors:** Li Yang, Cheng-Lun Na, Shiyu Luo, David Wu, Simon Hogan, Taosheng Huang, Timothy E. Weaver

**Affiliations:** 1 Perinatal Institute, Division of Neonatology, Perinatal and Pulmonary Biology, Cincinnati Children’s Hospital Medical Center, Cincinnati, OH, 45229-3039, USA; 2 Division of Human Genetics, Cincinnati Children’s Hospital Medical Center, Cincinnati, OH, 45229-3039, USA.; 3 Division of Allergy and Immunology, Cincinnati Children’s Hospital Medical Center, Cincinnati, OH, 45229-3039, USA

## Abstract

Mitochondria synthesize select phospholipids but lack the machinery for synthesis of the most abundant mitochondrial phospholipid, phosphatidylcholine (PC). Although the phospholipid transfer protein Stard7 promotes uptake of PC by mitochondria, the importance of this pathway for mitochondrial and cellular homeostasis represents a significant knowledge gap. Haploinsufficiency for Stard7 is associated with significant exacerbation of allergic airway disease in mice, including an increase in epithelial barrier permeability. To test the hypothesis that Stard7 deficiency leads to altered barrier structure/function downstream of mitochondrial dysfunction, Stard7 expression was knocked down in a bronchiolar epithelial cell line (BEAS-2B) and specifically deleted in lung epithelial cells of mice (Stard7^*epi∆*/*∆*^). Stard7 deficiency was associated with altered mitochondrial size and membrane organization both *in vitro* and *in vivo*. Altered mitochondrial structure was accompanied by disruption of mitochondrial homeostasis, including decreased aerobic respiration, increased oxidant stress, and mitochondrial DNA damage that, in turn, was linked to altered barrier integrity and function. Both mitochondrial and barrier defects were largely corrected by targeting Stard7 to mitochondria or treating epithelial cells with a mitochondrial-targeted antioxidant. These studies suggest that Stard7-mediated transfer of PC is crucial for mitochondrial homeostasis and that mitochondrial dysfunction contributes to altered barrier permeability in Stard7-deficient mice.

The mechanism(s) by which newly synthesized phospholipids are transferred from the ER through an aqueous environment to a specific target membrane is poorly understood. The Steroidogenic acute regulatory protein (StaR)-related lipid transfer (START) domain is a ~210 amino acid module that binds and transfers lipid molecules between donor and acceptor membranes^1^,^2^. There are 15 mammalian proteins that contain a START domain, including a subfamily of 4 proteins that preferentially transfers ceramide (Stard11) or phosphatidylcholine (Stard2/PC-TP, Stard10, and Stard7). Stard7, the focus of this study, was originally detected in a choriocarcinoma cell line (JEG-3) and subsequently in a range of tumor cell lines, as well as normal cells/tissues^3^,^4^. Knockdown of Stard7 was associated with altered trophoblast cell behavior, including decreased cell migration, proliferation, intercellular desmosomes, and phospholipid synthesis^5^; in addition, Stard7 knockdown in HepG2 cells was associated with ER stress, increased reactive oxygen species (ROS) and altered mitochondrial morphology^6^. Importantly, Stard7 was shown to specifically transfer PC to mitochondria^7^, raising the possibility that many of the cellular phenotypes associated with loss of Stard7 expression may be downstream of mitochondrial dysfunction.

The Stard7 mRNA transcript encodes a protein, Stard7-1, that contains a mitochondrial targeting sequence (MTS)^7^. Cleavage of the NH_2_-terminal domain containing the MTS yields Stard7-2, an isoform that also binds/transfers phosphatidylcholine (PC). Horibata and Sugimoto^7^ reported that Stard7-1, but not Stard7-2, facilitated transfer of PC to mitochondria in HEPA-1 cells and, recently, demonstrated that Stard7 is important for mitochondrial homeostasis^8^. Mitochondria can synthesize specific phospholipids, including phosphatidylglycerol, phosphatidylethanolamine (PE), and cardiolipin, but lack the enzymatic machinery to synthesize PC via the *de novo* or remodeling pathways^9^,^10^. PC is the major phospholipid species in mitochondria, comprising up to 45% of total mitochondrial phospholipids; further, PC is the major donor of linoleic acid for remodeling of cardiolipin^11^, a phospholipid dimer required for mitochondrial membrane organization and function of enzymes involved in oxidative phosphorylation^12^. Thus, import of PC, via transfer proteins or other processes such as membrane contacts^13^, is critical for mitochondrial function and homeostasis; yet little is known about the mechanism(s) underlying mitochondrial PC import or the impact of individual PC transfer pathways on cellular homeostasis and organ function.

To begin to address this knowledge gap, we generated global knockout mice for Stard7^14^. Stard7^−/−^ mice died at E11 while Stard7^+/−^ mice survived and were fertile. Unexpectedly, approximately 30% of adult Stard7^+/−^ mice developed pruritic skin lesions associated with marked Th2 inflammation and elevated IgE; moreover, Stard7^+/−^ mice mounted an exaggerated allergic airway response following allergen sensitization and challenge^14^. These results suggest that Stard7 is an important component of a novel protective pathway that modulates allergic responses at critical mucosal-environmental interfaces. In support of this hypothesis, Stard7 expression was decreased by 50% in the nasal epithelia of human patients with an acute asthma exacerbation^14^,^15^. Whether the pathogenesis of allergic airway disease is causally linked to mitochondrial dysfunction in Stard7-deficient cells remains unknown.

As much as 20% of the world’s population is affected by allergic disease^16^. Allergic asthma is a chronic inflammatory disorder characterized by infiltration of Th2 cells and eosinophils, increased mucus production and bronchial hyperresponsiveness^17^. It has been proposed that asthma is primarily a defect of epithelial barrier function that allows greater access of environmental allergens, microorganisms, and toxicants to airway tissues^18^,^19^. Importantly mitochondrial dysfunction, oxidative stress, and impaired barrier function have all been linked to asthma pathogenesis^20^,^21^,^22^,^23^,^24^. In this study, we tested the hypothesis that deficiency of Stard7 in airway epithelial cells perturbs mitochondrial homeostasis and that this event is linked to an increase in airway epithelial barrier permeability.

## Results

### Mitochondrial structure is altered in Stard7-deficient cells

Haploinsufficiency for Stard7 is associated with significant exacerbation of allergic airway disease, including increased airway epithelial barrier permeability^14^. Since a likely function for Stard7 involves targeted transport of PC to mitochondria^7^, the current study was designed to determine if Stard7 deficiency leads to mitochondrial dysfunction that, in turn, leads to altered barrier structure/function in Stard7-deficient mice. Expression of the two Stard7 isoforms, Stard7-1 and Stard7-2^4^, was first assessed in normal human bronchial epithelial cells immortalized with SV40T antigen (BEAS-2B cells). Western blotting of BEAS-2B cell lysates detected endogenous Stard7 as a band migrating with Mr~34 k, consistent with the predicted mass of Stard7-2 (Fig. S1a). Following transfection of BEAS-2B cells with Stard7 shRNAs, two cell lines were identified in which both Stard7 mRNA and protein were decreased by >90%. Transient transfection of knockdown cells (shRNA-1 cell line), with a shRNA-resistant construct encoding HA-tagged Stard7-1, demonstrated that most Stard7-1 protein (Mr~43 k) was converted to the smaller Stard7-2 isoform (Fig. S1b). Inhibition of mitochondrial function by CCCP resulted in a prominent increase in Stard7-1 and a decrease in Stard7-2. These results in BEAS-2B cells confirm similar findings in HEPA-1 cells and support the previously proposed model in which Stard7-1 is targeted to mitochondria, where the NH_2_-terminal peptide of ~75 residues is rapidly cleaved to generate Stard7-2^7^.

**Figure 1:**
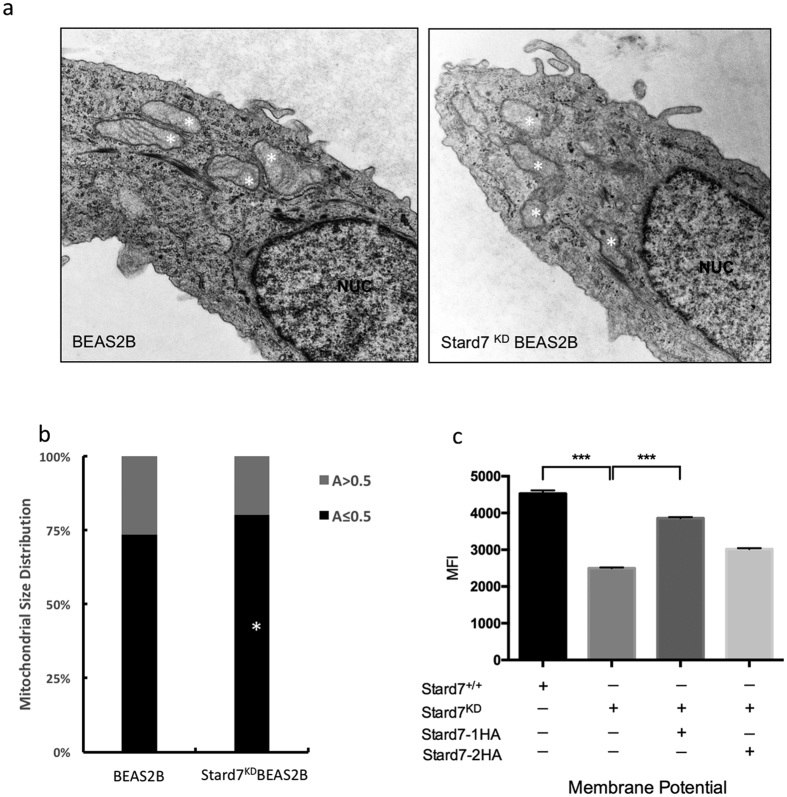
Stard7 deficiency is associated with altered mitochondrial structure in BEAS-2B cells. (**a**) Transmission electron microscopy of BEAS-2B and Stard7^KD^BEAS-2B cells detected smaller mitochondria and poorly defined cristae in knockdown cells compared to WT cells. NUC: nucleus. (**b**) Images of mitochondria labeled by TOMM22 antibody were collected from BEAS-2B (*n* = 47) and Stard7^KD^BEAS-2B cells (*n* = 48) for morphometric analyses of surface area (A). Stard7^KD^BEAS-2B cells had higher than expected numbers of smaller mitochondria (A ≤ 0.5 μm^2^) compared to BEAS-2B cells (*p = 0.0045). (**c**) Mitochondrial membrane potential (Δψm) was assessed after incubation with or without DilC_1_(5) for 30 minutes followed by flow cytometry. Stard7 deficiency was associated with decreased Δψm. BEAS-2B cells vs. Stard7^KD^BEAS-2B cells, ***p < 0.0001. Transient transfection of Stard7^KD^BEAS-2B cells with Stard7-1HA partially restored Δψm. Stard7^KD^BEAS-2B/Stard7-1HA vs. Stard7^KD^BEAS-2B cells, ***p < 0.0001. MFI, mean fluorescence intensity.

The effect of Stard7 deficiency on mitochondrial structure was assessed by analyses of BEAS-2B control (untransfected) and knockdown cells. Cristae were readily detected by transmission electron microscopy (TEM) in most mitochondria of control BEAS-2B cells but were less frequently observed in Stard7-deficient (shRNA-1 knockdown) cells (Fig. 1a), similar to recent findings in two liver cell lines^6^,^8^. To assess the impact of Stard7 deficiency on mitochondrial size, confocal images of control and knockdown cells were subjected to morphometric analysis of Tomm22^+^ compartments (Fig. 1b). Loss of Stard7 expression was associated with a significant increase in the number of mitochondria with surface area <0.5 μm^2^, p = 0.0045 (Fig. 1b). Mitochondrial membrane potential was assessed by staining control and knockdown cells with Di1C_1_(5) (MitoProbe) followed by flow cytometry (Fig. 1c). Accumulation of membrane-sensitive dye was decreased in knockdown cells, consistent with fewer mitochondria with active membrane potentials. Transfection of Stard7-1 resulted in partial correction of mitochondrial membrane potential whereas transfection of Stard7-2 did not significantly alter membrane potential. Altogether, mitochondrial size, membrane organization, and membrane potential were altered in Stard7-deficient BEAS-2B cells.

### Mitochondrial respiration is perturbed in Stard7-deficient cells

**Figure 2:**
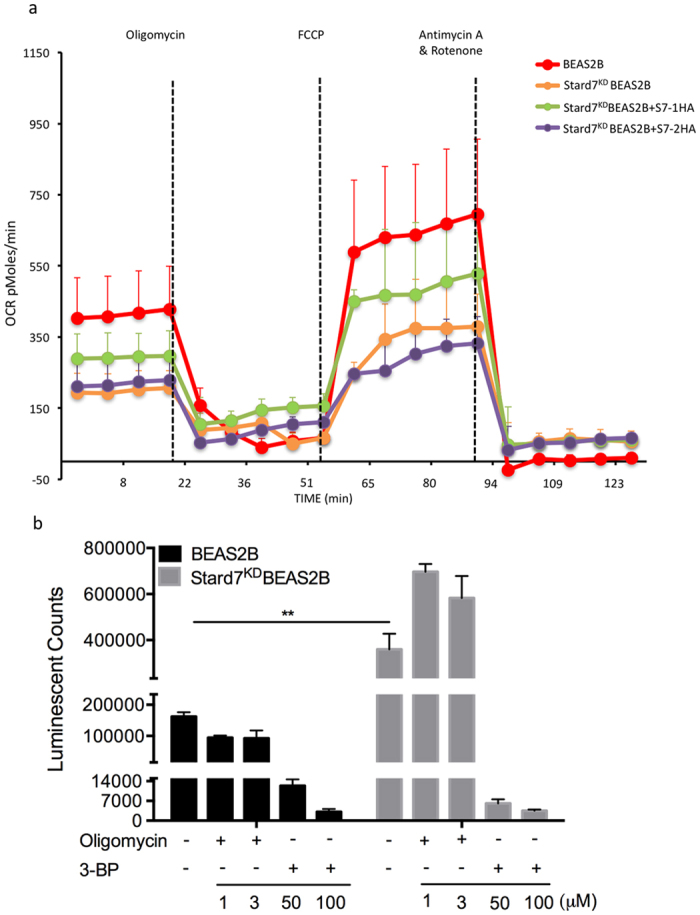
Stard7 deficiency is associated with altered mitochondrial function. (**a**) Mitochondrial OCR (pMoles/min) was measured under basal conditions and in response to the indicated mitochondrial inhibitors. The OCR was lower in Stard7^KD^BEAS-2B cells. Transient transfection of Stard7^KD^BEAS-2B cells with Stard7-1HA partially restored OCR (mean ± SEM, *n* = 5). (**b**) BEAS-2B and Stard7^KD^BEAS-2B cells were treated with or without the indicated concentration of 3-BP or oligomycin for one hour and cellular ATP levels were determined. Data represent the averages of three independent experiments (mean ± SEM). BEAS-2B cells vs. Stard7^KD^BEAS-2B cells, **p = 0.0078. OCR: oxygen consumption rate. FCCP: carbonyl cyanide p-trifluoromethoxyphenylhydrazone. 3-BP: 3-bromopyruvate.

The potential importance of Stard7 for mitochondrial respiration was assessed by measuring the oxygen consumption rate (OCR) in control and knockdown cells, using the Seahorse Extracellular Flux (XF24) Analyzer (Fig. 2a). Stard7 deficiency resulted in a decrease in baseline respiration that was partially restored by transient transfection of Stard7-1 but not Stard7-2. Maximal respiration was also affected in knockdown cells, resulting in a dramatic decrease in spare respiratory capacity that was partially rescued by Stard7-1, similar to recent findings in HEPA-1 cells^8^. Incomplete rescue of basal and maximal respiration by Stard7-1 (as well as mitochondrial membrane potential, Fig. 1c) is likely related to transfection efficiency of ~50% in these experiments. Since Stard7-1 contains a mitochondrial targeting sequence (MTS) that is not present in Stard7-2, these results are consistent with the conclusion that mitochondrial targeting of Stard7 is critical for maintenance of mitochondrial respiration.

The primary function of mitochondria is the production of ATP. Decreased mitochondrial respiration in Stard7-deficient cells was unexpectedly associated with an increase in cellular ATP concentration (Fig. 2b). Inhibition of ATPase synthase (oligomycin treatment) resulted in decreased cellular ATP in WT BEAS-2B cells but not in knockdown cells, consistent with elevated non-mitochondrial respiration in the absence of Stard7 (Fig. 2b), similar to results in HEPA-1 cells^8^. Inhibition of glyceraldehyde-3-phosphate dehydrogenase (3-bromopyruvate treatment) depleted ATP content in both WT and knockdown cells (Fig. 2b). These results confirm that glycolysis contributes to maintenance of ATP levels in Stard7-deficient cells.

### Stard7 deficiency is associated with increased ROS and mtDNA damage

**Figure 3:**
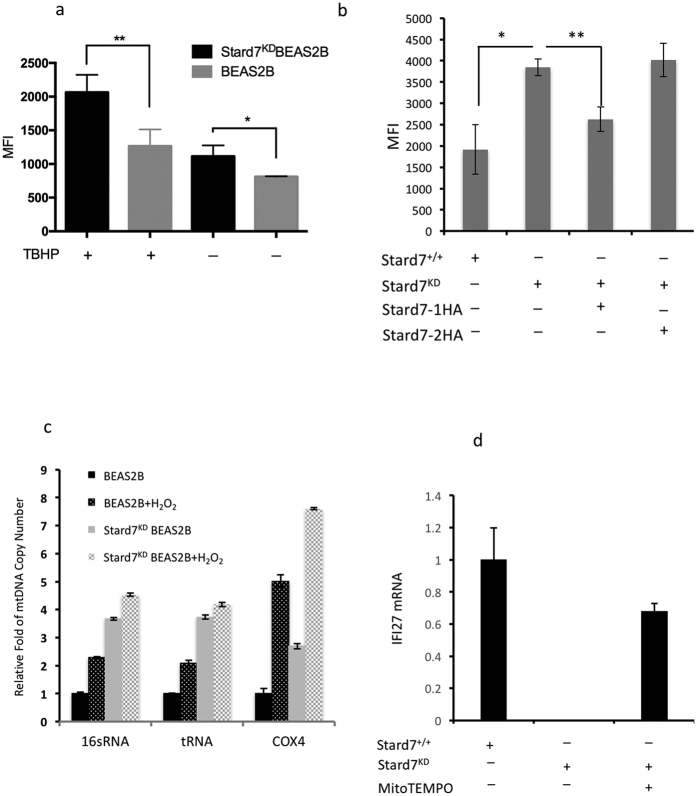
Stard7 deficiency leads to excessive generation of ROS and mitochondrial DNA damage. (**a**) BEAS-2B and Stard7^KD^BEAS-2B cells were incubated with or without DCFDA (20 μM) and/or TBHP (25 μM) to induce oxidant stress and cultured for 3 hours. ROS were analyzed by flow cytometry. Data are expressed as MFI (mean ± SEM, *n* = 3); *p = 0.032, BEAS-2B cells vs. Stard7^KD^BEAS-2B cells; **p = 0.0186, TBHP-treated BEAS-2B cells vs. TBHP-treated Stard7^KD^BEAS-2B cells. (**b**) Knockdown cells were transiently transfected with Stard7-1HA or Stard7-2HA and ROS levels assessed as described in panel a. *p = 0.0111, Stard7^KD^BEAS-2B cells vs. BEAS-2B cells; **p = 0.0072, Stard7-1HA transfected Stard7^KD^BEAS-2B cells vs. Stard7^KD^BEAS-2B cells. (**c**) Relative mitochondrial DNA copy numbers were determined by qRT-PCR using primers for mitochondrial genes (16 sRNA, tRNA and Cox4) or a nuclear gene (actin). (**d**) Expression of the oxidant–sensitive gene interferon alpha inducible protein 27 (IFI27) was assessed in BEAS-2B and Stard7^KD^BEAS-2B cells by qRT-PCR before and after treatment with MitoTEMPO. MFI: mean fluorescent intensity. DCFDA: dichlorofluorescin diacetate; TBHP: tert-butyl Hydroperoxide.

Altered mitochondrial homeostasis can result in increased generation of ROS and mtDNA damage, in addition to other pathologic changes. To determine if Stard7-deficiency was associated with altered generation of ROS, control and knockdown cells were incubated with 2′,7′-dichlorofluorescin diacetate (DCFDA), which is deacetylated to a non-fluorescent compound by cellular esterases. Generation of ROS results in oxidation to highly fluorescent 2′,7′-dichlorofluorescein (DCF) that can be detected by flow cytometry. Basal ROS were significantly elevated in knockdown cells compared to control cells (Fig. 3a). Oxidant challenge with tert-butyl hydroperoxide (TBHP) resulted in elevated ROS in both control and knockdown cells, although oxidant burden was significantly higher in Stard7-deficient cells. Transfection of knockdown cells with Stard7-1, but not Stard7-2, decreased ROS to levels comparable to those in control cells (Fig. 3b). Excessive accumulation of ROS can damage mitochondrial DNA leading to changes in mitochondrial DNA conformation and gene expression. Mitochondrial DNA exists primarily in a supercoiled conformation that is a relatively poor template for DNA amplification^25^; oxidative damage results in strand breaks and a relaxed DNA structure that is a better substrate for PCR. Results of qPCR analyses for 3 mitochondrial genes (16 S RNA, t RNA, and Cox4) in control and knockdown cells indicated that Stard7 deficiency was associated with a significant increase in copy number for all 3 mitochondrial gene targets (Fig. 3c). Treatment with H_2_O_2_ significantly increased DNA amplification in control cells as expected but, with the exception of Cox4, only modestly increased DNA amplification in knockdown cells. Moreover, expression of an oxidant-sensitive gene, interferon alpha inducible protein 27 (IFI27), was dramatically downregulated in knockdown cells and upregulated in response to treatment with the mitochondrial-targeted antioxidant, MitoTEMPO (Fig. 3d). These findings suggest that Stard7 deficiency leads to elevated mitochondrial ROS that, in turn, results in significant perturbation of mitochondrial DNA structure. In support of this hypothesis, sequencing of the mitochondrial genome confirmed that oxidative stress in Stard7-deficient cells was associated with an increase in sequence variations compared to WT BEAS-2B cells (Table 1).

### Paracellular permeability is increased following Stard7 knockdown

**Figure 4:**
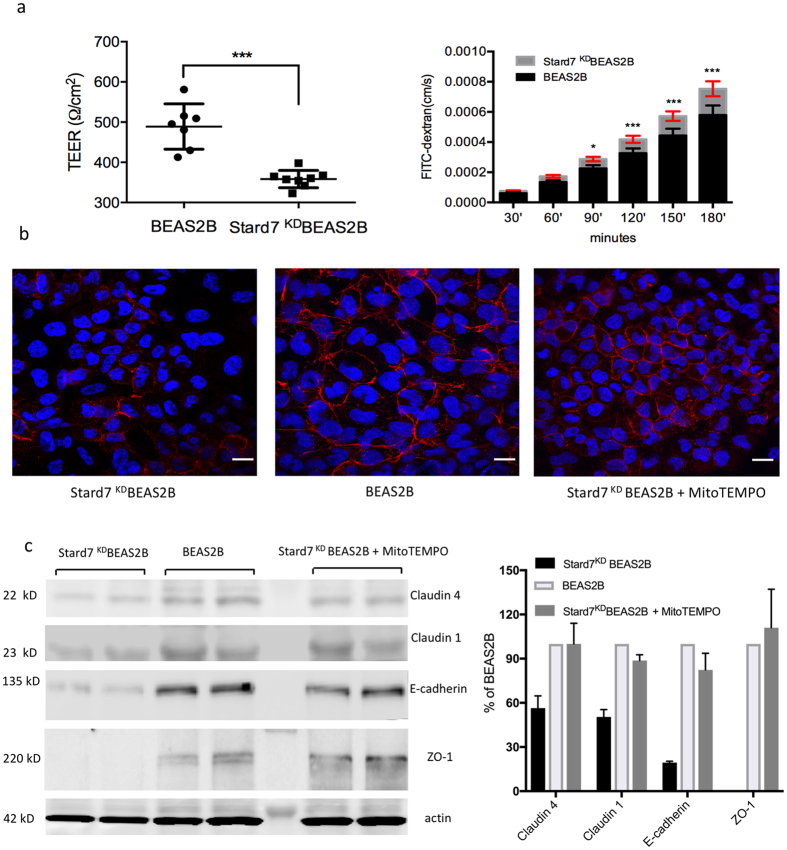
Stard7 deficiency increases epithelial barrier permeability. (**a**) The effect of Stard7 deficiency on BEAS-2B cell monolayer integrity was assessed by measuring transepithelial electrical resistance (TEER, left panel). The effect of Stard7 deficiency on the transepithelial permeability was determined by measuring apical to basolateral diffusion of FITC-dextran at the indicated time points (right panel). Data are expressed as mean ± SEM, *n* = 3; *p < 0.05, ***p < 0.0001, BEAS-2B cells vs. Stard7^KD^BEAS-2B cells. (**b**) Expression of the adherens junction protein E-cadherin was assessed by immunofluorescent staining of BEAS-2B cells and Stard7^KD^BEAS-2B cells cultured in the presence or absence of MitoTEMPO. Scale bars = 50 μM. (**c**) Apical junction protein expression was assessed in BEAS-2B cells and Stard7^KD^BEAS-2B cells, cultured in the presence or absence of MitoTEMPO. Equal numbers of cells from each experimental group were aliquoted in duplicate, cultured for 48 h, and 20 μg protein from each cell lysate analyzed by SDS-PAGE/western blotting (left panel). Protein bands were quantitated by densitometry, the data normalized to actin, and mean values expressed as percent of BEAS-2B control cells (right panel).

We next assessed the effect of Stard7 deficiency on epithelial barrier permeability. Confluent cultures of BEAS-2B cells undergo squamous differentiation to form a selectively permeable epithelial monolayer in culture. Transepithelial resistance (TEER) was significantly decreased and paracellular permeability to FITC-dextran was increased in monolayers of knockdown cells compared to control cells (Fig. 4a); further, cell-cell adhesion, as visualized by staining for E-cadherin, (Fig. 4b), was disrupted in knockdown cells. Consistent with a leaky monolayer, expression of the tight junction proteins claudin-1, claudin-4 and ZO-1 was decreased in Stard7-deficient cells (Fig. 4c). Importantly, treatment of knockdown cells with a mitochondrial-targeted antioxidant, MitoTEMPO, substantially restored E-cadherin expression (Fig. 4b) as well as claudin-1, claudin-4 and ZO-1 expression (Fig. 4c). Collectively, analyses in BEAS-2B cells suggest a model in which loss of Stard7 expression leads to mitochondrial dysfunction and oxidative stress that, in turn, promotes increased epithelial barrier permeability.

### Targeted deletion of Stard7 in lung epithelial cells is associated with altered mitochondrial structure and function *in vivo*

**Figure 5:**
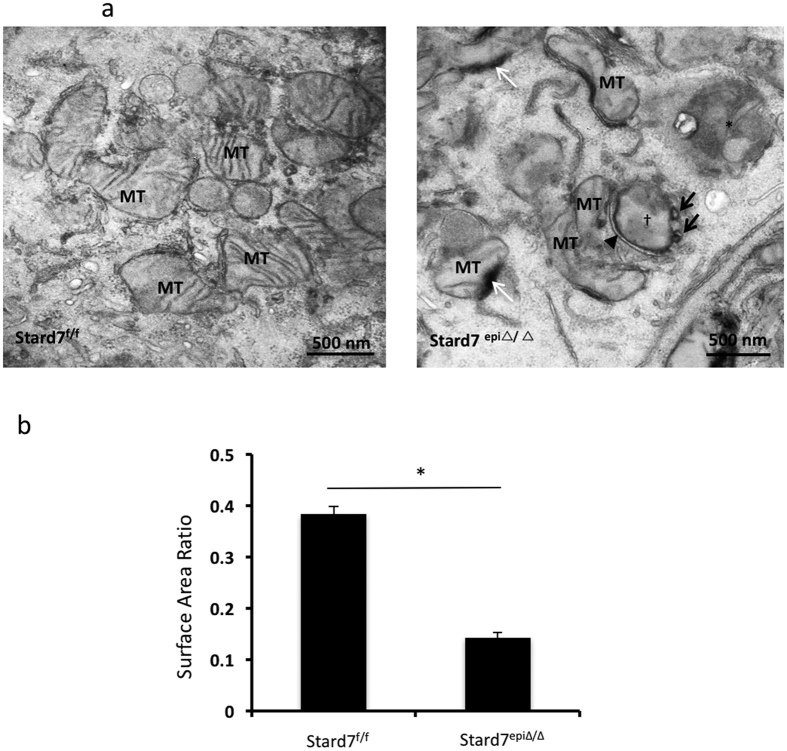
Altered mitochondrial structure in airway epithelial cells of Stard7^epiΔ/Δ^ mice. (**a**) Airway sections obtained from lungs of 4-month-old Stard7^f/f^ and Stard7^epiΔ/Δ^ mice were examined by electron microscopy. Stard7^epiΔ/Δ^ mice exhibited mitochondrial abnormalities in bronchiolar epithelial cells, including poorly defined mitochondrial matrix, reduction in number of cristae, increased lipid accumulation juxtaposed to outer limiting membrane of mitochondria (white arrow), formation of mitochondrial blebs (black arrow), and mitochondrial disintegration. Formation of an isolation membrane (small arrow) encircling a mitochondria (cross) and autophagosomes (asterisk) containing mitochondrial fragments were pronounced in some Stard7^KO^ cells. (**b**) Electron micrographs of mitochondria in ciliated bronchiolar epithelial cells, acquired from 4-month-old Stard7^f/f^ and Stard7^epiΔ/Δ^ mice, were used for morphometric analysis. There was a significant reduction of mitochondrial cristae in ciliated bronchiolar epithelial cells of Stard7^epiΔ/Δ^ mice compared to Stard7^f/f^ mice, **p < 0.001; MT = mitochondria.

As a first step toward evaluating Stard7 function *in vivo*, we generated Stard7^f/f^ mice and crossed them to Shh-Cre driver mice to generate animals with targeted deletion of Stard7 in epithelial cells of the lung (Stard7^epi∆/∆^, Fig. S2). Loss of Stard7 expression in lung epithelial cells was confirmed by immunocytochemistry (Fig. S3). Ultrastructural analyses of airway epithelial cells detected lipid inclusions and mitochondria with decreased numbers of cristae in the subapical region of ciliated cells of Stard7^epi∆/∆^ mice compared to Stard7^f/f^ controls (Fig. S4). Higher power magnification revealed multiple mitochondrial abnormalities in Stard7-deficient cells, including lipid accumulation juxtaposed to the outer mitochondrial membrane (Fig. 5a, white arrows), formation of mitochondrial blebs (Fig. 5a, black arrows) and isolation membrane (Fig. 5a, arrowhead), and autophagosomes containing mitochondrial fragments (Fig. 5a, asterisk). Morphometric analyses of mitochondria in ciliated bronchiolar cells from Stard7^f/f^ and Stard7^epi∆/∆^ mice identified a >60% decrease in the surface area ratio of cristae in Stard7-deficient cells (Fig. 5b). These results indicate that targeted disruption of Stard7 expression in lung epithelial cells *in vivo* leads to profound changes in mitochondrial morphology.

**Figure 6:**
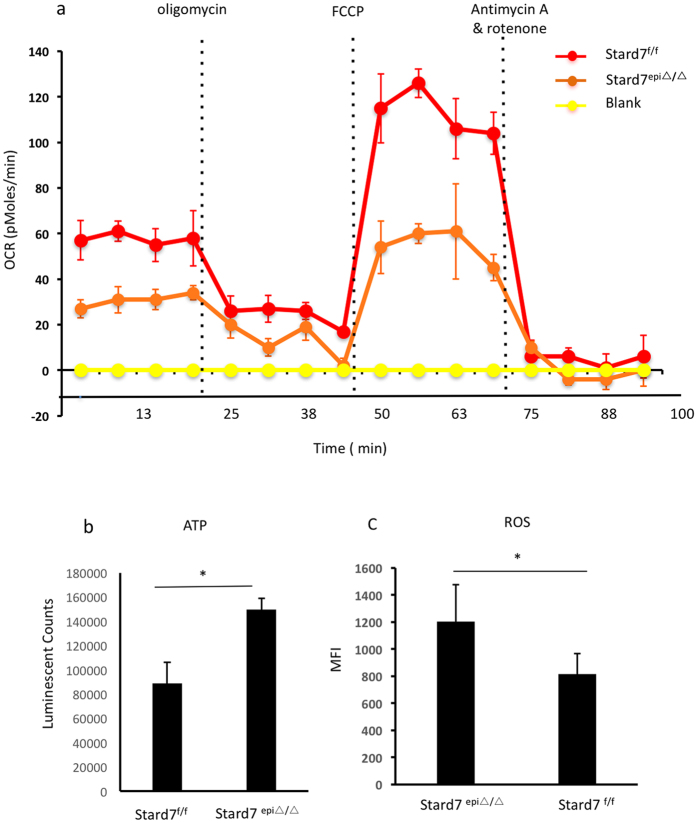
Impact of Stard7 deficiency on OCR, ATP, and ROS in lung epithelial cells. (**a**) Lung epithelial cells were isolated from 4-month-old Stard7^f/f^ and Stard7^epi∆/∆^ mice and OCR (pMoles/min) measured under basal conditions and in response to the indicated mitochondrial inhibitors. OCR was lower in lung epithelial cells from Stard7^epi∆/∆^ mice (mean ± SEM, *n* = 5). (**b**) Cellular ATP levels were assessed in isolated epithelial cells. Data are presented as mean ± SEM, *n *=* *3. There was a significant increase of ATP in lung epithelial cells of Stard7^epi∆/∆^ compared to Stard7^f/f^ mice, *p = 0.0115. (**c**) Isolated lung epithelial cells were incubated with or without DCFDA and analyzed by flow cytometry. ROS levels were significantly increased in lung epithelial cells of Stard7^epi/∆∆^ mice compared to Stard7^f/f^ mice, *p = 0.0193. Data is presented as MFI (mean ± SEM, *n* = 3).

To determine if altered mitochondrial structure was accompanied by changes in function, epithelial cells were isolated from lungs of Stard7^f/f^ and Stard7^epi∆/∆^ mice and subjected to mitochondrial stress test. Both resting and maximal respiration were decreased in Stard7-deficient cells (Fig. 6a) but cellular ATP content was maintained (Fig. 6b). Analyses of cellular ROS in isolated lung epithelial cells revealed increased oxidative stress in cells from Stard7^epi∆/∆^ mice compared to Stard7^f/f^ control mice (Fig. 6c). Taken together, these results indicate that targeted deletion of Stard7 leads to disruption of mitochondrial homeostasis in lung epithelial cells of Stard7^epi∆/∆^ mice, similar to findings in BEAS-2B cells (this study) and HEPA-1 cells^8^.

### Stard7 deficiency is associated with altered epithelial barrier integrity *in vivo*

**Figure 7:**
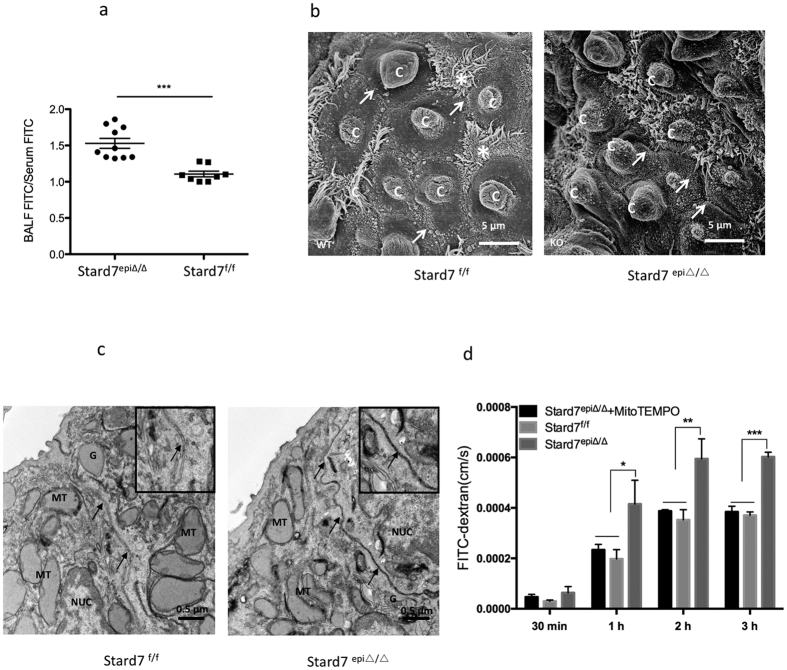
Altered epithelial barrier structure and function in Stard7^epi∆/∆^ mice. (**a**) Epithelial barrier permeability was assessed by tail vein injection of FITC-albumin, followed by quantitation of FITC in BALF and serum from Stard7^f/f^ and Stard7^epi∆/∆^ mice. Results are expressed as BALF FITC/Serum FITC, *n* = 8–10 mice/group. Stard7^f/f^ mice vs. Stard7^epi∆/∆^ mice, ***p = 0.0001. (**b**) SEM detected surface fractures between bronchiolar epithelial cells (asterisk) and club cells (C) in Stard7^epi∆/∆^ mice. (**c**) Fixed mouse lungs were cut into 1–2 mm lung slices, incubated with 2% lanthanum nitrate at room temperature overnight, and processed for electron microscopy. Penetration of electron dense lanthanum nitrate into the intercellular spaces (arrow) was detected in the airway epithelium of Stard7^epi∆/∆^ mice. G: Golgi, MT: mitochondria, NUC: Nucleus. (**d**) Tracheal epithelial cells were isolated from Stard7^epi∆/∆^ and Stard7^f/f^ mice and cultured until TEER >220/cm^2^. Cells were subsequently differentiated at an air-liquid interface for 3 weeks and treated with/without MitoTEMPO (5 μM) every other day. Paracellular flux of FITC-dextran was measured over a 3 hr period. Stard7^epi/∆∆^ vs. Stard7^epi∆/∆^ + MitoTEMPO, *p = 0.0311, **p = 0.0106, ***p < 0.001; Stard7^epi∆/∆^ vs. Stard7^f/f^, *p = 0.0201, **p = 0.0091, ***p < 0.001.

To determine the impact of altered mitochondrial structure and function on epithelial barrier integrity, mice were intravenously injected with FITC-albumin and barrier leak estimated by recovery of fluorescent label in bronchoalveolar lavage fluid (Fig. 7a). This analysis revealed that alveolar epithelial barrier permeability was significantly increased in Stard7^epi∆/∆^ mice compared to Stard7^f/f^ controls. Scanning electron microscopy of the surface of the bronchiolar epithelium detected tight cell-cell junctions among ciliated and club cells of 4-month-old Stard7^f/f^ mice (Fig. 7b, left panel). In contrast, surface fractures, consistent with disruption of cell-cell junctions, were detected in the bronchiolar epithelium of Stard7^epi∆/∆^ mice (Fig. 7b, right panel). Access of lanthanum nitrate to intercellular spaces in the bronchiolar epithelium of Stard7^epi∆/∆^ mice, but not Stard7^f/f^ mice, confirmed that paracellular permeability was increased in airways of Stard7-deficient animals (Fig. 7c). To further assess the effect of Stard7 deficiency on airway epithelial barrier function, tracheal epithelial cells (mTEC) were isolated from Stard7^epi∆/∆^ and Stard7^f/f^ mice and cultured at an air-liquid interface. Barrier leak, estimated from paracellular flux of FITC-dextran, was significantly increased in monolayers of Stard7-deficient cells (Fig. 7d). Importantly, incubation of Stard7-deficient cells with MitoTEMPO completely reversed the increase in epithelial barrier permeability. Collectively, these findings indicate that selective deletion of Stard7 in lung epithelial cells leads to mitochondrial dysfunction that, in turn, is associated with oxidant-mediated changes in epithelial barrier integrity and function.

## Discussion

Loss of Stard7 expression was associated with altered mitochondrial size and membrane organization in a human airway epithelial cell line and in lung epithelial cells of mice with targeted deletion of Stard7. Changes in mitochondrial structure were accompanied by perturbation of mitochondrial homeostasis, including decreased aerobic respiration, increased generation of ROS, and mitochondrial DNA damage. Impaired mitochondrial function was partially rescued by specific targeting of Stard7 to mitochondria. Mitochondrial dysfunction in Stard7-deficient epithelial cells was subsequently linked to increased epithelial barrier permeability *in vitro* and *in vivo*. These findings provide evidence that Stard7 is required for mitochondrial structure/function and suggest that Stard7-mediated PC transfer is important for mitochondrial homeostasis and that mitochondrial dysfunction associated with Stard7 deficiency can lead to altered epithelial barrier function.

In the present study, western blotting of airway epithelial cells detected Stard7 as a protein with Mr~34k (Stard7-2), with only a trace amount of Stard7-1 (Mr~43k), consistent with previous findings in a liver cell line^7^ and placental tissue/cells^26^. Generation of Stard7-2 likely occurs by proteolytic processing of Stard7-1 resulting in removal of a 75-amino acid leader sequence that harbors a MTS, predicted to be contained within residues 1–58^7^. Removal of the domain downstream of the MTS (~17 amino acids) likely occurs at the OMM, but the precise cleavage site(s) and the identity of the enzyme(s) involved in the cleavage event are not known. Rapid conversion of Stard7-1 to Stard7-2^7^ and localization of Stard7 to cytoplasm, plasma membrane and nucleus^26^,^27^ suggests that Stard7-2 may also have a non-mitochondrial function. Given the ability of Stard7-2 to bind and transport PC^7^, it is possible that Stard7-2 may facilitate exchange of PC among intracellular organelles other than mitochondria. The importance of this putative PC transport pathway and the regulation of PC transfer activity, by association with other proteins and/or post-translational modification(s) of Stard7-2, remain unknown.

Consistent with the ability of Stard7-1 to specifically transfer PC to mitochondria, Horibata, *et al*.^8^ recently demonstrated that PC species in isolated mitochondria were decreased following knockdown or knockout of Stard7 in HEPA-1 cells. Based on the complete absence of Stard7 in knockout cells, the change in mitochondrial PC content was less than expected, an outcome that likely reflects redundancy in mitochondrial PC transfer pathways. In this regard, phosphorylation of S110 by protein Kinase C was previously shown to mediate interaction of Stard2/PC-TP with the OMM, although it is not known if this interaction is coupled to PC transfer^28^. The ability of Stard10 to function as a mitochondrial PC transfer protein has not been tested. It is possible that PC transfer pathways may also involve phospholipid transfer proteins that are not members of the START domain family. Additionally, PC transfer may occur at ER-OMM contact sites referred to as mitochondrial associated membranes (MAM)^13^; however, although import of PS via MAMs has been studied in mammalian cells, comparable analyses for PC are lacking^9^,^10^. The consequences of mitochondrial PC deficiency are also poorly understood. In yeast, depletion of PC affected the import, folding, and integration of proteins into the OMM^29^: altered import of nuclear-encoded proteins into the mitochondria can lead to protein accumulation in the cytosol with consequent activation of stress response pathways^30^,^31^. Whether altered protein translocation contributes to mitochondrial dysfunction in Stard7-deficient cells remains to be determined. Overall, loss of PC associated with Stard7 deficiency likely contributed to changes in mitochondrial structure and function by altering membrane geometry and disrupting formation of supercomplexes required for mitochondrial respiration. In this regard Horibata, *et al*.^8^ showed that the formation and stability of supercomplexes was altered in Stard7 knockdown cells.

Disruption of mitochondrial respiration in Stard7-deficient cells did not negatively impact cellular ATP content; rather, ATP levels were maintained by glycolysis, although we cannot exclude the possibility that decreased energy consumption also contributed to an increase in ATP availability. Stard7 deficiency was associated with increased ROS in both BEAS-2B cells and isolated lung epithelial cells (this study) as well as HepG2 cells^6^. Oxidant stress has been linked to increased barrier permeability, in part, through disruption of apical junctional complexes ^AJC^ 32,33,34,35. AJC consist of tight junctions and underlying *adherens* junctions that regulate paracellular solute passage and cell-cell adhesion, respectively. In this study, increased ROS were associated with decreased expression of key tight junction proteins, including claudin-1, claudin-4, and ZO-1, and increased epithelial barrier permeability in Stard7-deficient cells. Expression of E-cadherin, an *adherens* junction protein critical for cell-cell adhesion, was also significantly perturbed and was substantially rescued by treatment of Stard7-deficient cells with a mitochondrial-targeted antioxidant. Further, MitoTEMPO treatment of airway epithelial cells from Stard7-deficient mice restored barrier function. Collectively, these findings suggest that Stard7 deficiency leads to increased mitochondrial ROS that, in turn, promote epithelial barrier leak. Importantly, mitochondrial dysfunction, oxidative stress and epithelial barrier dysfunction are strongly implicated in the pathogenesis of allergic airway disease^36^,^37^,^38^,^39^.

In summary, these studies demonstrate that loss of Stard7 expression is associated with altered mitochondrial structure and function, both *in vitro* and *in vivo*. Mitochondrial dysfunction arising from Stard7 deficiency was associated with elevated ROS and impaired epithelial barrier integrity; whether, barrier leak is sufficient to promote airway hyperreactivity or other aspects of the allergic airway phenotype in Stard7-deficient mice, remains to be determined. Lastly, given the relative abundance of cytosolic Stard7-2, we cannot exclude the possibility that loss of Stard7 expression may also affect cellular homoeostasis via a non-mitochondrial pathway(s).

## Methods

### Generation and characterization of Stard7^epi∆/∆^ mice

All experiments involving mice were performed in accordance with guidelines and regulations approved by the Institutional Animal Care and Use Committee of the Cincinnati Children’s Research Foundation (Cincinnati, OH). All mice were housed in a pathogen-free barrier facility in humidity- and temperature-controlled rooms on a 12:12 h light/dark cycle and were allowed access to food and water *ad libitum*. Stard7^epi∆/∆^ mice were generated by breeding three existing lines of mice, as illustrated in Fig. S3. Primers used for genotyping are listed in Table 2 (supplement). Antibodies used for western blotting or immunofluorescence microscopy are described in Table 3 (supplement).

### Knockdown of Stard7 in BEAS-2B Cells

BEAS-2B cells were grown and maintained in RPMI-1640 media with 5% heat-inactivated FBS. The sequence of the Stard7 shRNA (Sigma, TRCN0000151458) is listed in Table 4 (supplement). Lentiviral particles were prepared by the Viral Vector Core (Cincinnati Children’s Hospital Medical Center). Cells in 10-cm plates were transduced with viral particles (multiplicity of infection = 45-90) in 1 ml of RPMI-160 media containing 6 μg/ml of polybrene (Sigma, USA). After 24 h, conditioned media was removed and replaced with fresh RPMI-160 media. Cells were selected with 10 μg/ml puromycin (Sigma) for 1 month after viral transduction. Stard7 mRNA (Stard7 probe set from Integrated DNA Technologies, Hs.PT.58.20774759) and protein were assessed by qPCR and western blotting of Stard7 knockdown (Stard7^KD^) BEAS-2B cells.

### Generation of Stard7-1HA and Stard7-2HA Constructs

Stard7 constructs resistant to Stard7shRNA were generated by introducing silent mutations (bold letters) using the oligonucleotides listed in Table 4 (supplement). Site-directed mutagenesis of human Stard7 was performed according to the manufacturer’s protocol (QuikChange II XL Site-Directed Mutagenesis Kit, Agilent Technologies, Cat^#^ 200522) and verified by DNA sequence analysis. Primers used for Stard7-1HA and Stard7-2HA constructs are listed in Table 4 (supplement). An HA tag (YPYDVPDYA)^40^ was encoded in the downstream primer. The amplified Stard7-1HA and Stard7-*2*HA PCR fragments were cloned into the EcoV/XbaI sites of pcDNA3.1(+) vector (Invitrogen, Carlsbad, CA). Stard7^KD^BEAS-2B cells were transfected with pcDNA3.1-Stard7-1HA, pcDNA-Stard7-2HA or empty vector plasmid using X-tremegene HP DNA transfection reagent (Sigma-Aldrich, Cat^#^ 06366236001).

### Mitochondrial Respiration

Mitochondrial respiration was determined by measuring oxygen consumption rate (OCR) with a Seahorse XF24 Analyzer and the XF Cell Mito Stress Test Kit (Seahorse Bioscience, North Billerica, MA, USA). Briefly, BEAS-2B cells (1 × 10^5^ cells/well) or Stard7^KD^BEAS-2B cells were seeded onto 24-well XF-PS plates overnight. On the day of the assay, cell culture media was replaced with XF assay media (unbuffered Dulbecco’s modified Eagle’s medium supplemented with 11 mM glucose, 2 mL L-glutamax, and 1 mM sodium pyruvate). Basal OCR was measured over time followed by sequential addition of the mitochondrial inhibitors oligomycin (1 μM), carbonyl cyanide-p-trifluoromethoxyphenylhydrazon (3 μM), and antimycin A/rotenone (1 μM). ATP-linked respiration was derived from the difference between OCR at baseline and following oligomycin addition. The difference in OCR between antimycin A and oligomycin represented the amount of oxygen consumed due to proton leak. Maximal OCR was determined by subtracting the OCR after antimycin A addition from the OCR induced by carbonyl cyanide-p-trifluoromethoxyphenylhydrazone (FCCP). Lastly, the reserve capacity was calculated by the difference between maximal and basal respiration.

### Mitochondrial Membrane Potential

Mitochondrial membrane potential (Δψm) was assessed using the membrane potential sensitive cyanine dye DilC_1_(5) (MitoProbe, ThermoFisher, Cat^#^ M34151). The dye becomes concentrated in mitochondria with active membrane potentials leading to an increase in fluorescence intensity; disruption of Δψm significantly compromises DilC_1_(5) accumulation, resulting in a decrease of fluorescence intensity. BEAS-2B or Stard7^KD^BEAS-2B cells (2 × 10^5^/200 μl) were incubated with or without 25 nM DilC_1_(5) for 20 min at 37 °C, followed by 2 wash steps and analysis by flow cytometry (LSRII flow cytometer, BD Bioscience).

### Mitochondrial ROS Analysis

ROS were assayed using DCFDA, according to the manufacturer’s instructions (Abcam, Cat^#^ab113851). BEAS-2B or Stard7^KD^BEAS-2B cells (5 × 10^5^ cells/ml) were incubated with 20 μM DCFDA for 30 min at 37 °C, then analyzed by flow cytometry. Cells were also treated with TBHP (50 μM) to induce oxidative stress.

### Mitochondrial (mt) DNA Copy Number and Mutation Analyses

Total genomic DNA from 1 × 10^7^ BEAS-2B and Stard7^KD^BEAS-2B cells was isolated with QIAamp kit (Qiagen). The relative mtDNA copy numbers were measured by real-time PCR (ABI PRISM 7900) for mitochondrial genes (16sRNA, tRNA and Cox4) and normalized by simultaneous quantitation of nuclear DNA (actin). Primer sequences are listed in Table 4 (supplement). PCR was performed for 40 cycles with 10 ng DNA in a 25-μL reaction mixture using a SYBR green PCR master mix kit and 50 nmol forward and reverse primers. PCR cycling conditions included a 15-s denaturation step at 95 °C, 20-s annealing step at 60 °C, and 15-s extension step at 72 °C. A melting curve was performed for 20 min after the real-time PCR, and analysis was performed using the Dissociation Curve Software. The amplified products were denatured and reannealed at different temperatures to detect their specific melting temperatures. The threshold cycle number (Ct) values were determined in the same quantitative PCR run and results confirmed by a second run. Ct values were used as a measure of the copy number, and Ct value differences were used to quantify mtDNA copy number relative to β-actin, calculated as follows: relative copy number (Rc) = 2^∆CT^, where ∆CT = Ctβ-actin – CtmtDNA^41^.

To determine if elevated ROS were associated with increased frequency of mtDNA mutation, the mitochondrial genomes of BEAS-2B and Stard7^KD^BEAS-2B cells were compared (Table 1). mtDNA was amplified in a single PCR reaction, as previously described^42^ using primers F-2120 and R-2119 (Table 4, supplement) that specifically recognize mtDNA but not nuclear mitochondrial pseudo-genes. Amplified DNA was used for library preparation with the Nextera XT DNA Kit (Illumina). Sequencing was performed on the Illumina MiSeq platform (DNA Core Facility, Cincinnati Children’s Hospital Medical Center), and sequence reads ranging from 100 to 200 bp were quality filtered and processed using NextGENe software. The sequence error correction feature (condensation) was used to reduce false-positive variants and produce sample consensus sequence and variant calls. Alignment without sequence condensation was used to calculate the percentage of the mitochondrial genome with a depth of coverage of 1,000. Starting from quality FASTQ reads, the reads were quality filtered and converted to FASTA format. Filtered reads were aligned to the human mitochondrial sequence reference NC_012920.1, followed by variant calling. Variant heteroplasmy was calculated by NextGENe software as follows: Base heteroplasmy (mutant allele frequency %) = mutant allele (forward + reverse)/total coverage of all alleles C, G, T, and A (forward + reverse) 100^43^.

### Mouse Tracheal Epithelial Cell Isolation and culture

Isolated mouse tracheal epithelial cells were cultured at an air-liquid interface, as previously described^44^. Briefly, mTECs were isolated from the tracheas of 6-8 week old Stard7^f/f^ and Stard7^epiΔ/Δ^ mice and grown as submerged cultures on the apical surface of membrane inserts in transwell plates for 10 days. When TEER exceeded 220 Ω/cm^2^, the apical media was removed and mTECs were differentiated at an air-liquid interface for 3 weeks prior to analysis.

### Scanning Electron Microscopy (SEM)

Lungs from 4-5-month-old Stard7^epi∆/∆^ and Stard7^f/f^ mice were inflation-fixed with 2% paraformaldehyde, 2% glutaraldehyde, and 0.1% CaCl2 in 0.1 M sodium cacodylate buffer, pH 7.3 for 30 min on ice. Lung slices (1-2 mm thick) were incubated with 1% osmium (EMS, Hatfield, PA) and 1.5% potassium ferrocyanide (Sigma-Aldrich, St. Louis, MO) in 0.1 M, pH 7.3, for 2 hours, dehydrated in a graded series of alcohol, washed with hexamethyldisilazane (EMS, Hafield, PA), and air dried in a chemical fume hood for up to 2 days. Lung slices were mounted on specimen stubs and coated with palladium/gold using a Denton Vacuum Desk IV sputter coater (Denton Vacuum, Moorestown, NJ). Scanning electron images were acquired using a Hitachi field emission scanning electron microscope SU8010 (Hitachi High Technologies America, Inc., Clarksburg, MD) at 5 kV.

### Transmission Electron Microscopy

Lungs from 4–5-month-old Stard7^f/f^ and Stard7^epi∆/∆^ mice were fixed for transmission electron microscopy as described for SEM, with modifications to visualize defective airway cell junctions by incubation with the permeability tracer lanthanum nitrate. Following inflation fixation, lung slices were incubated with 2% lanthanum nitrate in fixative at room temperature overnight, followed by routine preparation for TEM^45^,^46^. Electron micrographs were collected using a Hitachi H-7650 TEM (Hitachi High Technologies America, Inc., Dallas, TX) equipped with an AMT transmission electron microscope charge-coupled device camera (Advanced Microscopy Techniques, Woburn, MA).

### Morphometric Analysis of mitochondria by Confocal Microscopy and TEM

Mitochondrial morphometry was determined by image analysis using a protocol modified from Koopman *et al*.^47^. Mitochondria of BEAS-2B cells were labeled with TOMM22 antibody and analyzed by confocal microscopy as previously described^46^. Acquired image stacks of labeled BEAS-2B cells were segmented by channel separation and thresholding to generate binary images. Subsets of labeled BEAS-2B cells were extracted and resampled at 0.5 μm sample distance from the original image stacks (sample distance 0.125 μm) for morphometric analysis. Binary images of labeled mitochondria were measured using the shape descriptor module in FIJI^48^.

Randomly selected ciliated bronchiolar epithelial cells obtained from the airways of 4-month-old Stard7^f/f^ and Stard7^epi∆/∆^ mice were chosen for mitochondrial morphometry at the EM level. To determine the size and shape of mitochondria, individual mitochondria were manually traced using a Wacom Intuos graphic tablet. Binary images of traced mitochondria were segmented by thresholding, followed by point counting, as described by Mülhfeld *et al*.^49^.

### Statistical Analyses

Data are expressed as mean ± SEM. Significant difference between groups were analyzed using Prism 5 software (GraphPad Software, San Diego, CA) by unpaired Student t test and ANOVA, unless otherwise noted. Statistical significance was set at *p ≤ 0.05, **p ≤ 0.01, and ***p ≤ 0.001.

## Additional Information

**How to cite this article:** Yang, L. *et al*. The Phosphatidylcholine Transfer Protein Stard7 is Required for Mitochondrial and Epithelial Cell Homeostasis. *Sci. Rep.* **7**, 46416; doi: 10.1038/srep46416 (2017).

**Publisher's note:** Springer Nature remains neutral with regard to jurisdictional claims in published maps and institutional affiliations.

## Supplementary Material

Supplementary Information

## Figures and Tables

**Table 1:** Mitochondrial DNA Mutations in Stard7^KD^BEAS-2B.

Nucleoid Position	Gene	Mutation Call	Heteroplasmy %
3992	NADH-ubiquinone oxidoreductase chain 1	c.686C > CT	49.06
4024	NADH-ubiquinone oxidoreductase chain 1	c.718A > AG	49.06
4587	NADH-ubiquinone oxidoreductase chain 2	c.118T > CT	46.88
5004	NADH-ubiquinone oxidoreductase chain 2	c.535T > CT	48.48
8269	Cytochrome oxidase II	c.684G > AG	51.85
9123	ATP synthase F0 subunit 6	c.597G > AG	53.12
10044	tRNA glycine	IVS1-15A > AG	50
12618	NADH dehydrogenase subunit 5	c.282G > AG	11.11
13105	NADH dehydrogenase subunit 5	c.769A > AG	46.43
13947	NADH dehydrogenase subunit 5	c.1611C > AC	9.01
14365	NADH dehydrogenase subunit 6	c.309C > CT	50
14582	NADH dehydrogenase subunit 6	c.92T > TC	47.37

Table depicting mutations in mitochondrial genes of Stard7^KD^BEAS-2B cells. A total of 12 mutations were found.
