# Supplementary material for: The Phosphatidylcholine Transfer Protein Stard7 is Required for Mitochondrial and Epithelial Cell Homeostasis
Source: Sci Rep. 2017 Apr 12;7:46416. doi: 10.1038/srep46416 (PMC5388865; doi:10.1038/srep46416)
Supplement: Supplementary Information [file srep46416-s1.pdf]

Supplementary Information

The Phosphatidylcholine Transfer Protein Stard7 is Required for Mitochondrial and Epithelial Cell Homeostasis

Li Yang (li.yang@cchmc.org)[1]

Cheng-Lun Na (cheng-lun.na@cchmc.org)[1]

Shiyu Luo (shiyu.luo@cchmc.org)[3]

David Wu (david.wu@cchmc.org)[2]

Simon Hogan (simon.hogan@cchmc.org)[2]

Taosheng Huang (taosheng.huang@cchmc.org)[3]

Timothy E. Weaver (tim.weaver@cchmc.org)[1*]

Perinatal Institute, [1]Division of Neonatology, Perinatal and Pulmonary Biology, [2]Division of Allergy and Immunology, and [3]Division of Human Genetics, Cincinnati Children's Hospital Medical Center, Cincinnati, OH 45229-3039

*Correspondence

Timothy E. Weaver, Ph.D.

Division of Pulmonary Biology, MLC7029

Cincinnati Children's Hospital Medical Center

3333 Burnet Avenue

Cincinnati, OH 45229-3039

Phone: 513-636-7223

Email: tim.weaver@cchmc.org

Fig S1

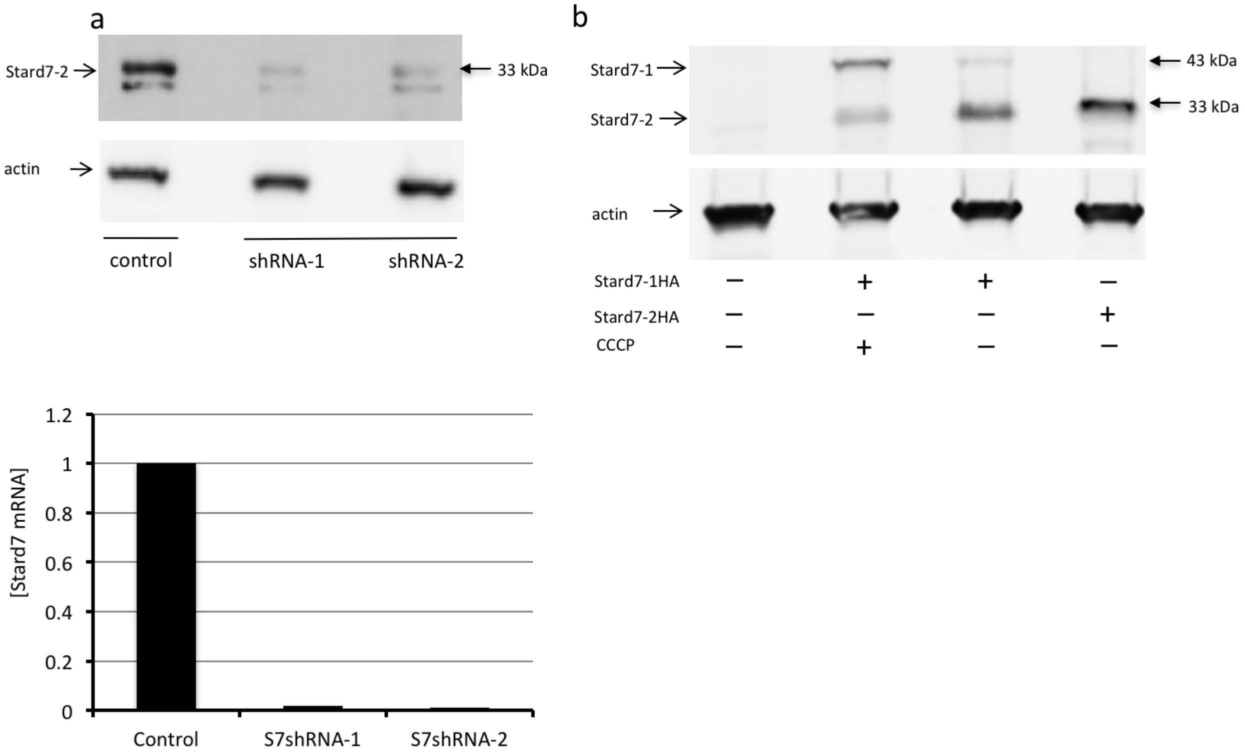

Fig S1. Stard7 protein and mRNA expression in BEAS-2B cells. (a) Endogenous Stard7 protein in BEAS-2B cells was assessed by western blotting before (control) and after transfection with Stard7 shRNA (top panel); Stard7 mRNA was assessed by qRT-PCR (bottom panel). Results are expressed as fold changes in Stard7 mRNA expression after normalizing to ß-actin. (b) Stard7^{KD}BEAS-2B cells (expressing Stard7shRNA) were transiently transfected with Stard7-1HA or Stard7-2HA with or without CCCP treatment, and cell lysates analyzed by western blotting with anti-HA antibody.

Fig.S2

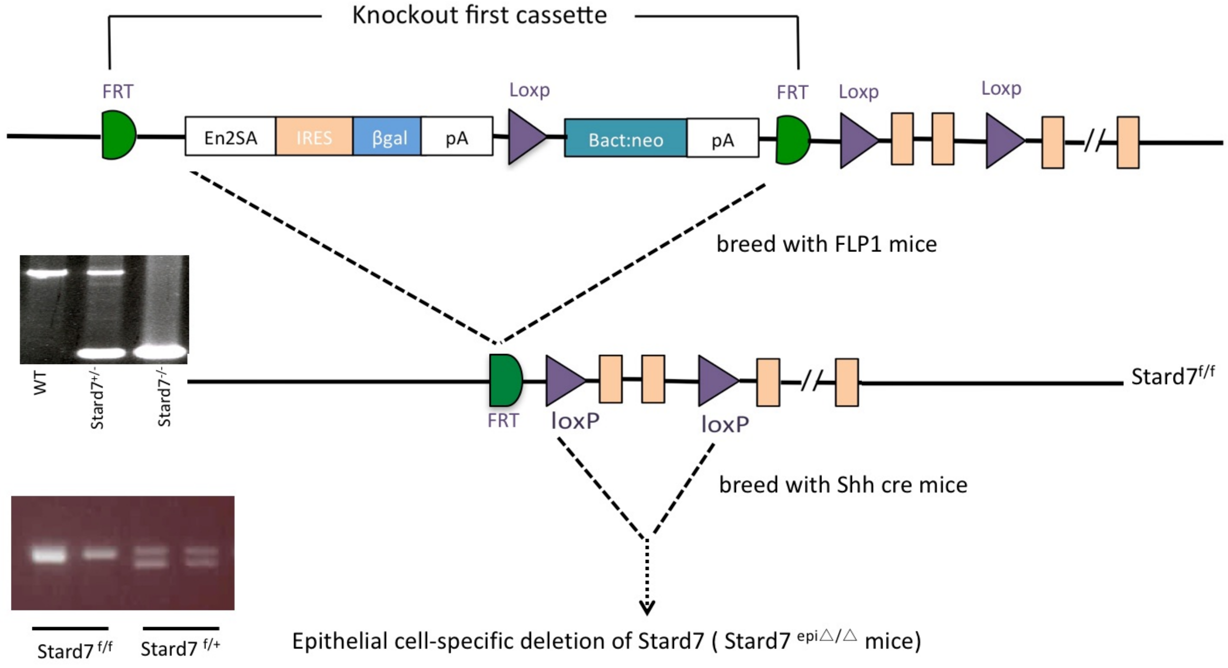

Fig S2. Generation of Stard7$^{epi\Delta/\Delta}$ mice. Schematic representation of epithelial cell-specific deletion of Stard7 in lungs of mice. The "Knockout first cassette" was removed by FLPI recombinase to generate Stard7$^{f/f}$ mice. Stard7$^{f/f}$ mice were bred with Shh-Cre driver mice to generate Stard7$^{epi\Delta/\Delta}$ mice**.** FLPI: Flippase.

Fig.S3

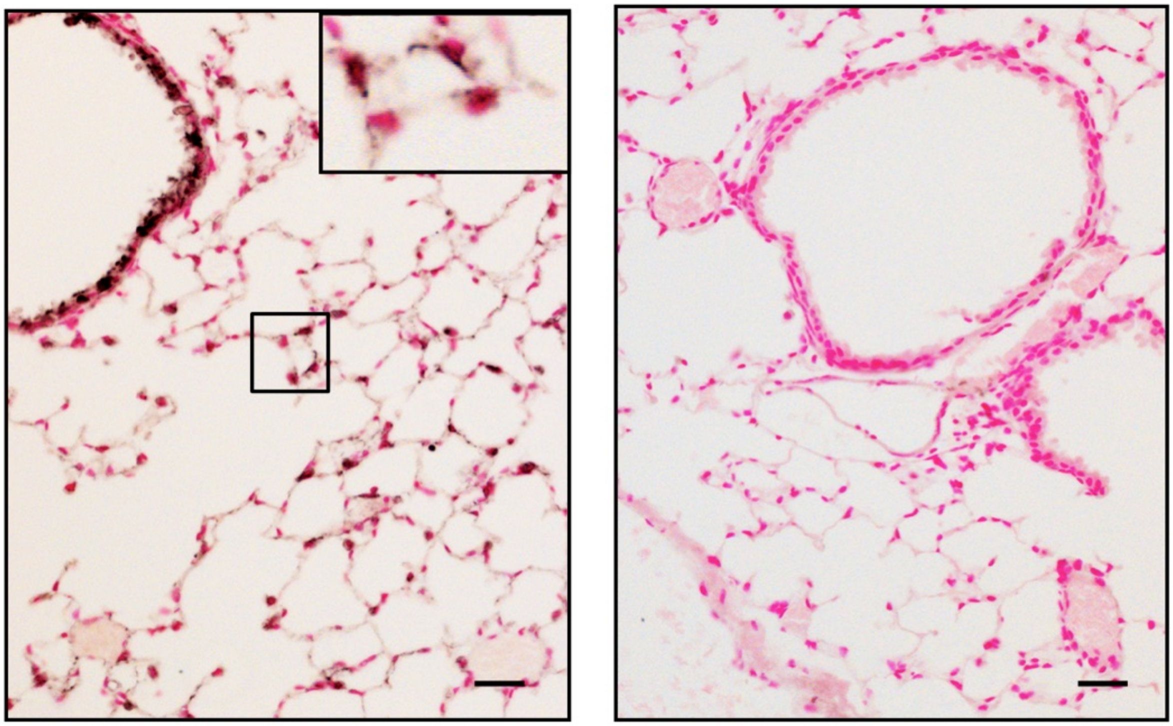

Fig S3. Stard7 protein expression in Stard7$^{epi\Delta/\Delta}$ mice. Immunohistochemical staining of Stard7 in lung sections from 8-week-old Stard7$^{f/f}$ and Stard7$^{epi\Delta/\Delta}$ mice. Scale bars = 25 μM.

Fig S4 Stard7 $^{f/f}$ Stard7 $^{epi\Delta/\Delta}$

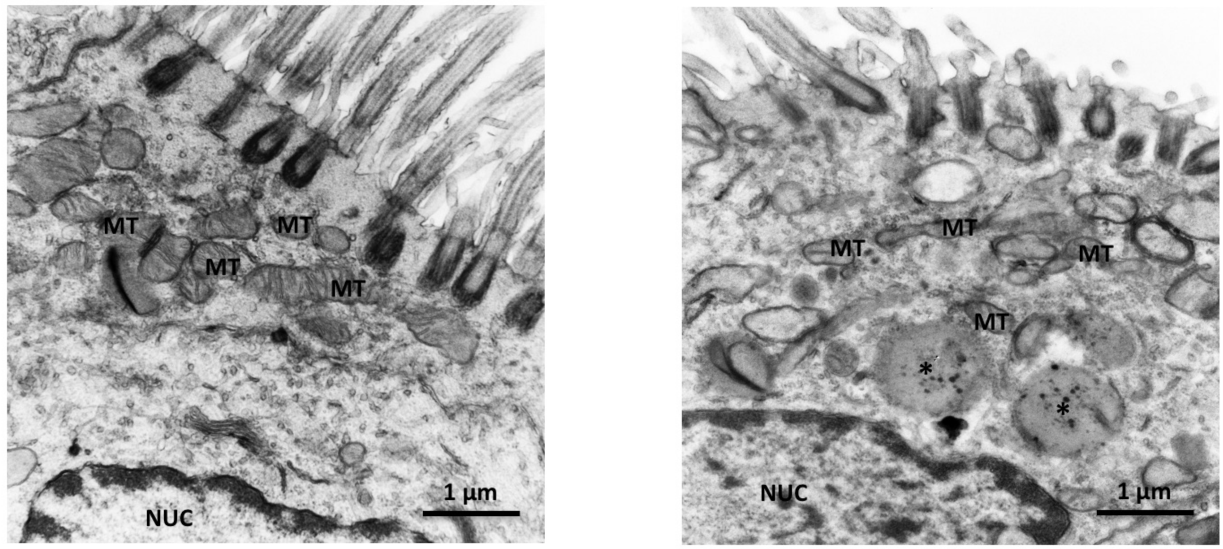

Fig S4. Mitochondrial abnormalities in ciliated bronchiolar epithelial cells of Stard7$^{epi\Delta/\Delta}$ mice. Mitochondria in ciliated bronchiolar epithelial cells of Stard7$^{epi\Delta/\Delta}$ mice contained fewer cristae compared to Stard7$^{f/f}$ mice. Pronounced lipid inclusions (asterisk) were detected in some ciliated bronchiolar epithelial cells. MT: mitochondria; NUC: nucleus.

SUPPLEMENTARY TABLES

Table 2: Mouse genotyping primers

Primers	Sequence
Stard7 null forward	5'-TCT ATA GTC GCA GTA GGC GG-3'
Stard7 WT forward	5'- GAT TTA GTG CCT CTA ACA GCA TGG GC-3'
Stard7 common reverse	5'-GAC TTT GGT TCG GGA GGG TAA TTC TC-3'
Shh-Cre forward	5'-GAT ATC TCA CGT ACT GAC GG-3'
Shh-Cre reverse	5'-TGA CCA GAG TCA TCC TTA GC-3'

Table 3: Antibodies

Antibody	Company	Catalog number	Titer
Actin	Seven Hills Bioreagents	LMAB-B4	1:2000
Claudin-1	Cell Signaling	4933	1:1000
Claudin-4	Abcam	ab53156	1:500
E-cadherin	Cell Signaling	3195s	1:1000
HA	Abcam	ab18181	1:1000
Tomm-22	*Novus Biologicals*	*H00056993-A0*	1:100
Stard7	Santa Cruz	sc-67855	1:500
ZO-1	Invitrogen	61-7300	1:500

Table 4: Oligonucleotide primers

Silent mutation primer	5'-AGA TCT ATT AAT GAG ATG AAG **CGC CTG GAG GAG ATG TCG AAC** ATG TTT CAG AGC TCT GGA GTC-3'
Stard7shRNA	5'- ccg gCG GTT GGA AGA AAT GTC AAA Tct cga gAT TTG ACA TTT CTT CCA ACC Gtt tttg-3'
Stard7-1HA forward	5'-CCG GAT ATC ATG CTC CCG CGG AGG CTG-3'
Stard7-2HA forward	5'-CCG GAT ATC ATG GCG GCG TTA GCC GGC-3'
Stard7 reverse	5'-CTA G TCT AGA TCA AGC GTA GTC TGG GAC GTC GTA TGG GTA AGC ATA CTC AAT CCG AGC-3'
ß-actin Forward	5'-TCA CCC ACA CTG TGC CCA TCT ACGA-3'
ß-actin reverse	5'-CAG CGG AAC CGC TCA TTG CCA ATGG-3'
16SRNA forward	5'-GCC TTC CCC CGT AAA TGA TA-3'
16sRNA reverse	5'-TTA TGC GAT TAC CGG GCT CT-3'
Cox4 forward	5'- CAA ACC TAC GCC AAA ATC CA-3'
Cox4 reverse	5'- GAA ATG AAT GAG CCT ACA GA-3'
tRNA forward	5'- CAC CCA AGA ACA GGG TTT GT-3'
tRNA reverse	5'-TGG CCA TGG GTA TGT TGT TA-3
F-2120	5'- GGA CAC TAG GAA AAA ACC TTG TAG AGA GAG-3'
R-2119	5'- AAA GAG CTG TTC CTC TTT GGA CTA ACA-3'
